# Platform Design Enabling Silver(III) Stabilization ─ The Uprise of Ag^III^CF_3_ Chemistry?

**DOI:** 10.1002/chem.202501606

**Published:** 2025-07-21

**Authors:** Luca Demonti, Daniel Joven‐Sancho, Miguel Baya, Noel Nebra

**Affiliations:** ^1^ Laboratoire Hétérochimie Fondamentale et Appliquée (LHFA) Université de Toulouse CNRS. 118 Route de Narbonne Toulouse 31062 France; ^2^ Instituto de Síntesis Química y Catálisis Homogénea (iSQCH) CSIC‐Universidad de Zaragoza Pedro Cerbuna 12 Zaragoza 50009 Spain

**Keywords:** Ag^III^ chemistry, Fluorine, Inverted ligand field (ILF), *Out‐of‐plane* coordination, Trifluoromethyl

## Abstract

Unlike its coinage metal counterparts, copper and gold, silver chemistry remains poorly developed and mostly restricted to the oxidation state +I, which embraces a large variety of coordination environments and types of ligand. Higher oxidation states, that is, +II and +III, are significantly less represented and predominantly adopt a square planar (*SP*‐4) geometry around the metal center. In particular, well‐defined d^8^ Ag^III^ compounds are scarce, and include only a handful of examples exhibiting *out‐of‐plane* silver coordination. This *Concept Article* summarizes the strategies known to stabilize Ag^III^, as well as the unusual electronic structures (inverted ligand field) often found in these compounds. The intriguing case study of the expansion in the coordination number in Ag^III^CF_3_ compounds will be highlighted.

## Graphical abstract

Oxidation state +III represents the highest known for silver compounds. This *Concept Article* critically analyzes the main strategies allowing for the authentication of silver(III) compounds, with special focus on Ag^III^CF_3_ species, that were discovered back in the 80′s and 90′s, but brought back to the scene very recently. They often display an inverted ligand field (ILF) electronic structure, which impacts their reactivity (i.e., inhibited axial nucleophilicity), and enables the expansion of the coordination number (up to 6) of *SP*‐4 Ag^III^ compounds.

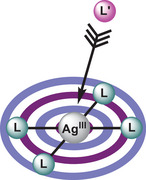

## 1 Introduction

As a lustrous and one of the noblest metals, silver has been extensively used by mankind since prehistoric times due to its rewarding properties, such as ductility and malleability, excellent thermal/electrical conductivity and reflectivity, low toxicity…^[^ ^1^, ^2^ ^]^ Akin to copper and gold, silver belongs to the metals of antiquity, being used in the manufacture of coins, ornaments, or jewelry.^[^ ^1^, ^2^ ^]^ Not only metallic silver, but its salts have found advantageous applications in the past. For instance, the disinfectant and microbiocidal power of AgNO_3_ is long known and exploited since Roman times in water treatment or bandages.^[^ ^3^ ^]^ In addition, silver finds utility nowadays in diverse industrial sectors including electronics, photovoltaic cells, photography, textile products, or medical imaging and materials.^[^ ^2^ ^]^

**Figure 1:**
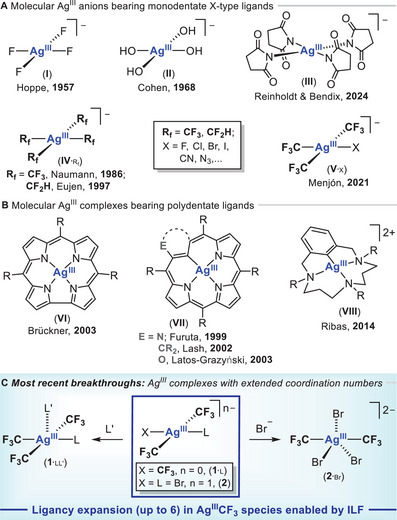
State of the art of molecular silver(III) compounds exhibiting the expected square planar (*SP*‐4) geometry for a d^8^ Ag^III^ ion bearing monodentate (**A**) or polydentate (**B**) ligands. (**C**) ***New avenues of development***: organosilver(III) compounds with extended coordination numbers.

Beyond the historical context, the coordination chemistry of silver is dictated by its ground state electronic configuration, [Kr]4d^10^5s^1^, but unlike copper and gold, it remains poorly developed and dominated by its prevailing oxidation state +I, that adopts a large variety of geometries and ligands.^[^ ^4^ ^]^ Silver redox chemistry is believed to mainly operate through 1*e* ^‐^ transitions.^[^ ^1a^ ^]^ Ag^I^ salts are primarily used as halide scavengers, Lewis acids, transmetallating agents, or oxidants for synthetic purposes.^[^ ^5^ ^]^ Although attainable, higher oxidation states (+II, +III) have shown propensity to get reduced, with some of these salts [*i.e*., Ag^II^F_2_,^[^ ^6^ ^]^ Ag^III^F_3_,^[^ ^7^ ^]^ [Ag^III^F_4_]^‐^ (**I**)^[^ ^8^ ^]^] being ranked amongst the strongest known oxidants.^[^ ^9^ ^]^ Nevertheless, although oxidation state +III constitutes the highest described for silver,^[^ ^9^ ^]^ good case examples of *SP*‐4 Ag^III^ compounds are long known (Figure 1 top and middle). This generally poor Ag^III^ stability may be driven into benefit, and remarkably, the capacity of organosilver(III) species to build C─CF_3_^[^ ^10^ ^]^ and C─heteroatom^[^ ^11^ ^]^ bonds via reductive elimination has been convincingly unveiled, what clearly denotes the current effervescence of the field.^[^ ^12^ ^]^ Seeking to circumvent the marked oxidizing behavior of high‐valent silver,^[^ ^9^ ^]^ three main strategies have been exploited granting Ag^III^ stabilization, namely:

- the preparation of Ag^III^ compounds bearing X‐type, strongly electronegative ligands (F, OH, NR_2_), typically anions [Ag^III^X_4_]^‐^ (**I‐III**) (Figure 1A, top);^[^ ^8^, ^13^, ^14^ ^]^
- the Ag^III^ encapsulation inside cavities created by polydentate ligand scaffolds, such as corroles (**VI**),^[^ ^15^ ^]^ confused (carba)porphyrines (**VII**),^[^ ^16^ ^]^ or tri‐aza‐arenes (**VIII**)^[^ ^11a^ ^]^ (Figure 1B); and
- the coordination of (per)fluoroalkyl (R_f_ = CF_3_, CF_2_H)^[^ ^17^ ^]^ ligands leading to the homoleptic anions [Ag^III^(R_f_)_4_]^‐^ (**IV^.^**_R__f_), or Ag^III^CF_3_ derivatives, [Ag^III^(CF_3_)_3_X]^‐^ (**V** ^.^_X_)^[^ ^11^, ^18^ ^]^ (Figure 1A, bottom).

This *Concept Article* seeks to collect and critically summarize the different tactics enabling the isolation and characterization of diverse Ag^III^ platforms, focusing on rare organosilver(III) entities displaying *out‐of‐plane* silver coordination. In addition to the aforesaid strategies leading to *“classical” SP*‐4 Ag^III^ species ―for a matter of space, only a selection of each kind will be briefly disseminated―, a handful of Sc‐XRD structurally characterized five‐ and six‐coordinate Ag^III^CF_3_ platforms will be highlighted herein together with their DFT bonding analyses pointing to the presence of an inverted ligand field (ILF).^[^ ^19^ ^]^ Although initially counterintuitive, this ILF electronic structure in *SP*‐4 Ag^III^ complexes makes the additional coordination of L‐type or X‐type ligands feasible thanks to the axial electrophilicity of the silver(III) center (Figure 1, bottom). To better rationalize this Ag^III^CF_3_ chemistry, the reader will be briefly introduced to concepts in the field such as penetration index,^[^ ^20^ ^]^ ILF,^[^ ^19^ ^]^ or *‘physical’* versus *‘formal’* oxidation state.^[^ ^21^ ^]^

## 2 Synthesis and Characterization of Well‐Defined Silver(III) Compounds

The chelating effect of polydentate ligands has been widely used in coordination chemistry to selectively bind metal cations and efficiently stabilize a wide range of inorganic complexes and organometallics.^[^ ^22^, ^23^ ^]^ In particular, *N*‐based macrocyclic ligands have seen widespread use owing to their appropriate flexibility/tunability in terms of ligand design, together with their peculiar geometries that bring along unique coordinating properties.^[^ ^24^ ^]^ More specifically related to the main topic of this article, and more generally, to high‐valent metal chemistry, the use of strongly donating tetradentate ligands represents a cornerstone to stabilize high oxidation state and electron‐deficient transition metal (TM) species.^[^ ^25^ ^]^ Of course, Ag^III^ chemistry is no exception and multiple compounds bearing tri‐ and tetra‐aza macrocyclic ligands, well‐suited to play a template role favoring *in‐plane* coordination, have been isolated and crystallographically characterized in the past. We disclose herein, to our taste, some of the most representative and iconic examples of Ag^III^ coordination compounds being conveniently stabilized by polydentate macrocyclic scaffolds (Sections 2.1–2.3). Following this, and complementarily, we describe the use of highly electronegative, monodentate ligands, either inorganic or (per)fluoroalkyl, as a successful strategy to stabilize other families of Ag^III^ complexes (Sections 2.4, 2.5).

### 2.1 Silver(III) Stabilization by Macrocyclic Encapsulation Using Poorly Flexible *N*‐Based Polydentate Ligands

**Figure 2:**
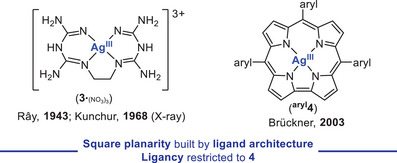
XRD‐Characterized *N*‐based Ag^III^ compounds **3** ^.^_(NO3__)__3_ and **^aryl^4** reported by Ray and Brückner, respectively.

To our knowledge, the first Ag^III^ complexes exclusively stabilized by *N*‐donor ligands, [Ag^III^(ethylendibiguanidinium)](X)_3_ (**3** ^.^_(X)__3_), were reported back in 1943 by Rây and Chakravarty, featuring an ethylendibiguanidinium ligand and different counterions (NO_3_, SO_4_, ClO_4_, OH). First geometrical insights were provided by the diamagnetism of **3**, that supports the presence of an *SP*‐4 Ag^III^ environment.^[^ ^26^ ^]^ The structure of **3** ^.^_(NO__3__)__3_ was elucidated 25 years later through Sc‐XRD analysis, revealing the ligand κ^4^‐coordination to Ag^III^ that adopts an ideal *SP*‐4 arrangement around silver (Figure 2, left).^[^ ^27^ ^]^ Later on, the syntheses of similar Ag^III^ compounds using biguanide‐based ligands were attempted by others,^[^ ^28^ ^]^ although with various reaction outcomes and without crystallographic characterization.

In 2003, a small array of five [*meso*‐triarylcorrolato]silver(III) complexes of type **^aryl^4** (Figure 2, right; aryl = Ph, *p*‐tolyl, *p*‐MeO‐C_6_H_4_, *p*‐NO_2_‐C_6_H_4_, 3,5‐di‐MeO‐C_6_H_3_) were prepared by Brückner and coworkers via disproportionation, by reacting the corresponding triarylcorroles with three equivalents of AgOAc in warm pyridine.^[^ ^29a^ ^]^ The Ag^I^‐to‐Ag^III^ oxidation proceeds through the Ag^I^ insertion into the macrocycle that facilitates the 2*e* ^‐^ oxidation with concomitant reduction of two‐thirds of sacrificial metal.^[^ ^29a^ ^]^ Once again, the resulting compounds proved diamagnetic and were characterized by NMR spectroscopy. Furthermore, XRD analysis on single crystals confirmed the square planarity around Ag^III^ imposed by the corrolato ligand, which becomes slightly saddled, thus providing the stabilization required to isolate the high‐valent, Ag^III^ compounds. This is supported by the shorter Ag^III^─N bond distances (av. 195 pm) and the smaller cavity size observed in the [*meso*‐tris‐*p*‐tolylcorrolato]silver(III) compound ***^p^* ^‐tolyl^4** compared to the ones typically found in porphyrin‐based Ag^II^ counterparts (av. Ag^II^─N bond length of 209 pm),^[^ ^29b^ ^]^ differently from Ag^III^‐*N*‐confused porphyrins case examples where the Ag^III^─N bond length remains comparable (av. 206 pm; see below).^[^ ^31^, ^32^, ^33^ ^]^ Following this report, related corrole‐based Ag^III^ complexes have been reported by Paolesse,^[^ ^33^ ^]^ Sankar,^[^ ^34^ ^]^ Kar,^[^ ^35^ ^]^ and Ghosh,^[^ ^36^ ^]^ all displaying the prototypical *SP*‐4 geometry brought by the rigidity imparted by the ligand of choice.

### 2.2 Organosilver(III) Compounds Stabilized by Rigid Macrocyclic Ligands

**Figure 3:**
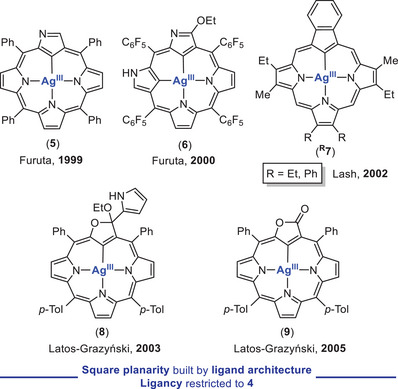
First examples of porphyrin‐based Ag^III^ complexes featuring mono *N*‐confused porphyrins (NCP) (top left), doubly NCP (top center), carbaporphyrins (top right) and *O*‐confused porphyrins (bottom).

Another ligand class with proven ability to enhance the stability of Ag^III^ complexes is constituted by the *N*‐confused porphyrins (NCP). In this family of ligands, one of the coordinating nitrogen atoms is replaced by a carbon atom belonging to the pyrrole core, thus becoming an X_3_L‐type of ligand. Such ligand modification affects drastically the properties of the resulting macrocycle, increasing its basicity and donicity, what enhances the stability of the resulting organosilver(III) compounds. Indeed, NCPs merge the best of corrole (trianionic nature) and porphyrin (bigger cavity size) rings, thus better accommodating the Ag^III^ ion inside the cavity. The first *N*‐confused porphyrin Ag^III^ complex (**5**; Figure 3, top left) was obtained by Furuta and coworkers in 1999 upon mixing AgOAc and the corresponding free‐base NCP.^[^ ^30^ ^]^ In line with the diamagnetism of this air‐stable *N*‐confused tetraphenylporphyrin argentate(III) complex **5**, its Sc‐XRD determined structure exhibits only minimal distortion from an ideal *SP*‐4 geometry. The suitability of NCPs to induce Ag^III^ stabilization instigated and resulted in independent contributions by the groups of Furuta and Latos‐Grazyński,^[^ ^32^, ^33^ ^]^ and notably, studies by the latter led to pioneering insights on the feasibility of Ag^III^‐mediated cross‐coupling reactions.^[^ ^12^, ^33^ ^]^ One year later, a remarkable contribution by Furuta and coworkers saw the light, and the organosilver(III) compound **6** was synthesized in similar fashion by using a doubly *N*‐confused porphyrin ligand (Figure 3, top center).^[^ ^37a^ ^]^

Other types of carbon‐donor macrocyclic ligand architectures enabling Ag^III^ stabilization are conceivable. In this sense, another relevant ligand modification was reported by Lash and coworkers, who isolated an array of Ag^III^ complexes of type **^R^7** (R = Et, Ph; Figure 3, top right). This novel porphyrin‐based ligands, namely carbaporphyrins, arise from the replacement of one pyrrole by a cyclic all‐carbon motif, a 2‐indenyl derived fulvene ring in the current case.^[^ ^39^, ^40^ ^]^ Later on, Latos‐Grazyński and coworkers demonstrated that *O*‐confused porphyrins are excellent candidates to achieve the synthesis and isolation of Ag^III^ complexes bearing an acetal (**8**) or a lactone (**9**) core within the ligand backbone (Figure 3, bottom).^[^ ^44^, ^45^ ^]^

### 2.3 Organosilver(III) Compounds Stabilized by Flexible Macrocyclic Polydentate Ligands

While rigid macrocyclic ligands proved highly effective for the isolation and characterization of different Ag^III^ platforms, the stabilization of high‐valent silver species using flexible polydentate cyclic ligands proved considerably more challenging. After the seminal work on stable Ag^III^ complexes by Rây,^[^ ^26^ ^]^ some groups sought to stabilize Ag^III^ compounds by coordinating the 1,4,8,11‐tetra‐aza‐macrocycle (cyclam).^[^ ^42^ ^]^ In this sense, Barefield and Mocella investigated the synthesis of Ag^III^‐cyclam complexes by electrochemical oxidation of the corresponding Ag^II^‐cyclam precursors, or alternatively, by chemical oxidation using NOClO_4_.^[^ ^42a^ ^]^ Despite the absence of crystallographic studies, the diamagnetic properties of the assumed Ag^III^ complexes were substantiated by using NMR spectroscopy, magnetic susceptibility measurements, and by the lack of EPR response.

**Figure 4:**
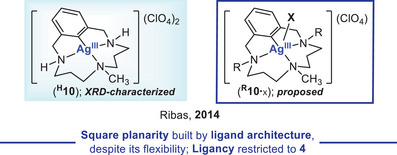
Square planar σ‐aryl‐Ag^III^ complex **^H^10** isolated and characterized by Ribas (left), along with the elusive square pyramidal aryl‐Ag^III^‐X intermediate **^R^10** ^.^_X_ (R = H, Me) proposed in C─heteroatom couplings enabled by Ag^I^/Ag^III^ redox cycles (right).

In 2014, Ribas and coworkers achieved the isolation and full characterization of a diamagnetic Ag^III^ complex **^H^10** stabilized by an aryl‐tri‐aza‐macrocycle pertaining to the pyclen family {pyclen = 3,6,9,15‐tetraazabicyclo[9.3.1]pentadeca‐1(15),11,13‐triene} (Figure 4, left).^[^ ^11a^ ^]^ These macrocyclic ligands had proven effective in the stabilization of high‐valent complexes with different metals,^[^ ^43^ ^]^ in particular organocopper(III) species,^[^ ^43a–d^ ^]^ thus making them suitable candidates to explore reminiscent organosilver(III) chemistry. Interestingly, **^H^10** has been obtained via oxidative addition of the aryl‐halide precursor to AgClO_4_. Despite the flexibility of the macrocyclic tri‐aza‐aryl ligand compared to the porphyrin‐like ones presented so far, **^H^10** crystallizes in an almost ideal *SP*‐4 geometry. With **^H^10** at hand, the authors successfully demonstrated its ability to mediate C─heteroatom bond forging reactions (including the challenging aromatic fluorination) through two‐electron Ag^I^/Ag^III^ redox catalysis, and invoked the participation of elusive square pyramidal aryl‐Ag^III^‐X species of type **^R^10** ^.^_X_ (R = H, CH_3_; Figure 4, right) as key intermediates on the basis of DFT studies.^[^ ^11a^ ^]^

The same group further investigated the Ag^III^‐catalyzed aryl─Nu bond‐forming reaction (with Nu being an *N*‐, *C*‐, or *O*‐based nucleophilic partner, Cl, or Br).^[^ ^44^ ^]^ To this end, an aminoquinoline skeleton has been employed, acting simultaneously as a directing group and a template for Ag^III^ stabilization. Although the crystallographic characterization of an organosilver(III) intermediate was lacking, the intermediacy of *SP*‐4 σ‐aryl‐Ag^III^ compounds formed through oxidative addition of the aryl halide to the silver(I) precursor has been invoked built on the template structure of aminoquinolines, previous work of the group,^[^ ^11a^ ^]^ and mass spectrometry analyses. By opposing the previous σ‐aryl‐Ag^III^ platform **^R^10^.^**_X_, the participation of an assumed square pyramidal (*SPY*‐5) catalytic intermediate has been discarded, and instead, the key R.E. step seems to occur from *SP*‐4 σ‐aryl‐Ag^III^‐X fragments.

### 2.4 Other Silver(III) Compounds Stabilized by *N*‐Donor and *O*‐Donor Ligands

**Figure 5:**
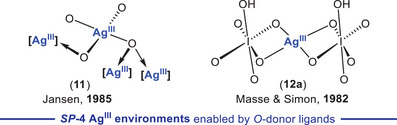
Simplified depictions of the crystallographically determined square planar polymeric structures of Ag_2_O_3_ (**11**, left) and [Ag^III^(HIO_6_)_2_]^5‐.^8xH_2_O (**12a** ^.^8xH_2_O, right). For clarity, H_2_O molecules are omitted.

A common strategy to enhance the chances of accessing the higher oxidation states of TMs relies on the preparation of anions bearing strongly electronegative ligands, particularly fluorides (details shown in Section 2.5 dedicated to this and also to (per)fluoroalkyl ligands), oxides, or hydroxides.^[^ ^9^ ^]^ Indeed, the oxo ligand (O^2‐^) presents ideal characteristics to stabilize high oxidation states metals, and thus, extreme oxidation states of early and middle TMs are often supported by oxo ligands.^[^ ^45^ ^]^ In line with the renowned oxo‐wall,^[^ ^46^ ^]^ the situation drastically changes for the preparation of stable late TM oxides, which remain underrepresented and typically tend to decompose. With respect to the silver case, Ag^I^ oxide (Ag_2_O) is commercially available and thermally stable, but susceptible to hydrolysis.^[^ ^47^ ^]^ Silver oxides in higher oxidation state are markedly unstable species leading to Ag_2_O easily.^[^ ^48^ ^]^ However, Ag_2_O_3_ (**11**) has been isolated and characterized.^[^ ^49a^ ^]^ This molecular solid, isostructural with its gold analogue (Au_2_O_3_),^[^ ^50^ ^]^ decomposes over ‐20 °C by O_2_ extrusion and crystallizes in nearly ideal *SP*‐4 Ag^III^ environment giving rise to a 3D polymeric network enabled by the bridging oxo ligands (Figure 5, left, shows the Sc‐XRD determined structure of **11** as originally depicted by the authors).^[^ ^49a^ ^]^ Other mixed valence silver oxides, such as AgO (Ag^I^Ag^III^O_2_)^[^ ^49b^ ^]^ or Ag_3_O_4_ (Ag^II^Ag_2_^III^O_4_),^[^ ^49c^ ^]^ have also been reported, all displaying similar Ag^III^ coordination environment.

Hydroxide ligands are equally well‐suited to stabilize highly oxidized metals. The preparation of the [Ag^III^(OH)_4_]^‐^ anion (**II** in Figure 1) in electrolysed basic AgO solutions has been argued long ago.^[^ ^13^ ^]^ Despite **II** has never been isolated nor structurally characterized, its trapping using KIO_3_ leads to [Ag^III^(HIO_6_)_2_]^5‐^ (**12a**; Figure 5, right).^[^ ^51^ ^]^ Complex **12a** and its related one, [Ag^III^(H_2_TeO_6_)_2_]^5‐^ (**12b**), are attainable by chemical methods.^[^ ^52^ ^]^ As might be anticipated, Sc‐XRD studies on **12a** confirmed the expected *SP*‐4 geometry around Ag^III^ with both HIO_6_ behaving as bidentate ligands.^[^ ^51^ ^]^ Nevertheless, these Ag^III^ anions **12a,b** are exclusively stable in alkaline medium, and accordingly, they find application as suitable oxidants in organic synthesis.^[^ ^53^ ^]^

On the other side, in contrast to the easy access to Ag^II^‐pyridine derivatives,^[^ ^54^ ^]^ the existence of Ag^III^ species bearing monodentate *N*‐donor ligands remained highly questioned until very recently.^[^ ^14^ ^]^ The formation of [Ag^III^(py)_4_](ClO_4_)_2_(NO_3_) by oxidation of AgNO_3_ in the presence of pyridine has been claimed, but unfortunately, this is still controversial owing to insufficient characterization data.^[^ ^55^ ^]^ To our knowledge, the first undeniable proof regarding the efficient stabilization of Ag^III^ by simple *N*‐donor ligands has been recently reported by Reinholdt and Bendix, who succeeded in the isolation and characterization of [Ag^III^(succinimide)_4_]^‐^ (**III** in Figure 1) by oxidizing the corresponding homoleptic Ag^I^ anion, or alternatively, by electrocrystallization.^[^ ^14^ ^]^ Sc‐XRD analysis on **III** shows a *SP*‐4 geometrical arrangement having an av. Ag^III^─N bond distance of 200.9 pm, similar to the ones found in the related porphyrin and carbaporphyrinoid systems commented above.

### 2.5 Fluoride and (Per)Fluoroalkyl Ligands. From Square Planarity to Extended Coordination Number

**Figure 6:**
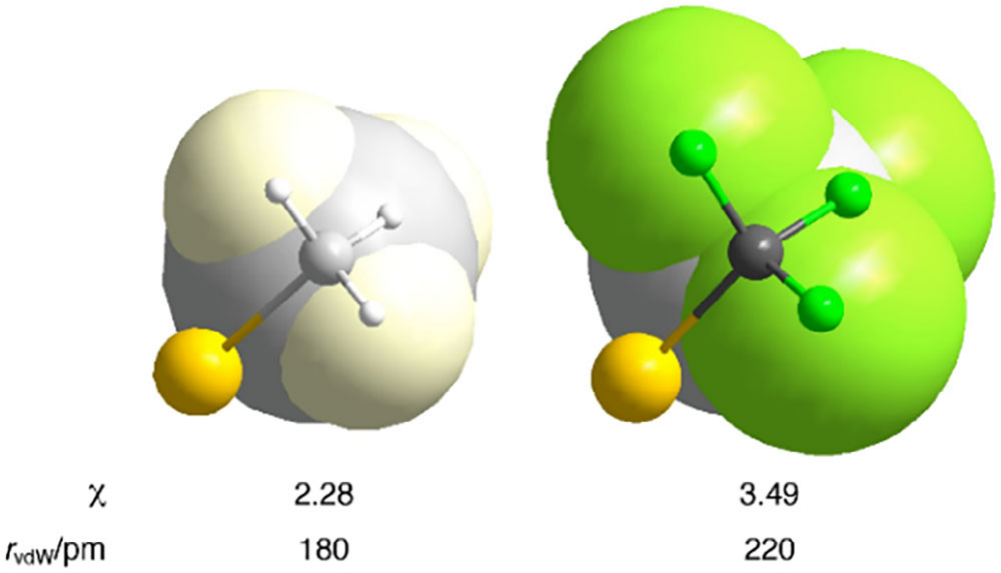
Comparative representation of the CH_3_ versus CF_3_ ligands in terms of size (*r*_vdW_ = effective van der Waals radius) and electronegativity (***χ***; on Pauling scale). Image created and shared by B. Menjón; reproduced with permission of Wiley‐VCH GmbH from the original source (B. Menjón and coworkers, “The Trifluoromethyl Group in Transition Metal Chemistry”, *Eur. J. Inorg. Chem*. **2012**, *2012*, 4945–4966).^[^ ^57a^ ^]^ © Copyright [**2012**] Wiley‐VCH GmbH. All rights reserved, including rights for text and data mining and training of artificial intelligence technologies or similar technologies.

Fluorine constitutes one of the best‐suited ligands to stabilize the highest oxidation state of TMs owing to its remarkable small size and high electronegativity. On the other hand, it is well established that replacing the hydrogen atoms by fluorine in organic motifs, for instance in a methyl group, drastically impacts the properties of a given organic skeleton.^[^ ^56^ ^]^ Keeping on this CH_3_/CF_3_ analogy, the trifluoromethyl group is considerably more electronegative than the methyl one (2.28 vs. 3.49 on the Pauling scale), while closer to an *iso*propyl group in terms of size (Figure 6).^[^ ^57^ ^]^ These critical changes make the CF_3_ (and related perfluoroalkyl ligands) optimal candidates for supporting high oxidation states of TMs, and particularly, the oxidation state +III for silver.

#### 2.5.1 Four‐Coordinate Ag^III^ Compounds

The simplest silver(III) fluoride, [Ag^III^F_4_]^‐^ (**I** in Figure 1),^[^ ^8a^ ^]^ was prepared by Hoppe in 1957 and later crystallized by others using different countercations,^[^ ^8^ ^]^ systematically showing the prototypical *SP‐*4 geometry consistent with a four‐coordinate d^8^ anion. It is to be noted that no binary salt with heavier halides (Cl, Br, I) has been reported so far. In fact, some reports by Schulz and Hargittai theoretically underlined the intrinsic instability of the heavier trihalides, Ag^III^X_3_ (X = Cl, Br, I), pointing to the key role of the fluoride ligands in the Ag^III^ stabilization.^[^ ^58^ ^]^ Besides Ag^III^‐fluorides, organosilver(III) compounds bearing fluoroalkyl ligands (R_f_ = CF_2_H, CF_3_) of the types [Ag^III^(R_f_)_n_X_4‐n_]^‐^ (n = 2–4)^[^ ^11^, ^17^, ^18^, ^63^, ^64^ ^]^ and Ag^III^(CF_3_)_3_L (**1** ^.^_L_),^[^ ^61^ ^]^ have been reported. Interestingly, the extensive evaluation of both families of compounds allowed to rationalize their relative stabilities. These are firstly influenced by the fluorination degree of the alkyl ligand, as clearly exemplified by the thermal stabilities of the two Ag^III^ homoleptic anions, [Ag^III^(R_f_)_4_]^‐^ [R_f_ = CF_2_H (**IV** ^.^_CF__2__H_), CF_3_ (**IV** ^.^_CF__3_)], that were first isolated and characterized by Eujen^[^ ^17a^ ^]^ and Naumann,^[^ ^17b^ ^]^ respectively. Whereas [Ag^III^(CF_2_H)_4_]^‐^ is stable up to 121 °C,^[^ ^17a^ ^]^ [Ag^III^(CF_3_)_4_]^‐^ starts to decompose at 188 °C.^[^ ^17c^ ^]^ Compounds of general formula [Ag^III^(CF_2_H)_n_X_4‐n_]^‐^ seem to be generally unstable,^[^ ^17^, ^59^ ^]^ while the related trifluoromethyl derivatives [Ag^III^(CF_3_)_n_X_4‐n_]^‐^ display considerable stabilities, mostly enabling their authentication and handling.^[^ ^11^, ^17^, ^18^, ^60^ ^]^ The homoleptic Ag^III^ anion bearing CH_3_ ligands, [Ag^III^(CH_3_)_4_]^‐^,^[^ ^62a^ ^]^ and related compounds [Ag^III^(CF_3_)_3_R]^‐^ (R = Me, Et, ^n^Bu, aryls),^[^ ^62b^ ^]^ have been detected in situ through mass spectrometry techniques by Koszinowski and coworkers, although their stability is certainly compromised.

**Figure 7:**
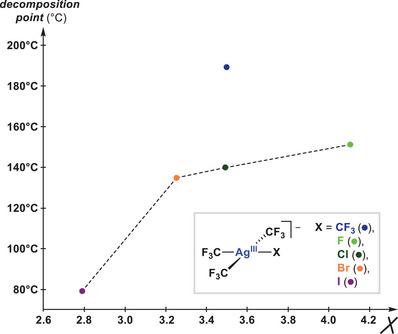
Schematic representation of Menjón's comparative study on the stability of [PPh_4_][Ag^III^(CF_3_)_4_] (**IV** ^.^_CF__3_) versus the series of halide‐Ag^III^ complexes [PPh_4_][Ag^III^(CF_3_)_3_X] (**V** ^.^_X_; with X = F, Cl, Br, I) as a function of the Pauling electronegativity scale (***χ***) of the X‐type ligand involved.

**Figure 8:**
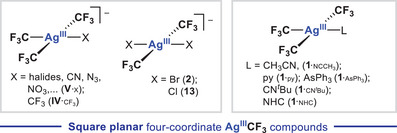
Representative examples of anionic (left) and neutral (right) square planar Ag^III^(CF_3_)_x_ (x = 2, 3) platforms reported back in the 90′s by Naumann and Eujen, and more recently, by Menjón, Shen, Nebra, and Baya.

In the same vein, the halide series of anions [Ag^III^(CF_3_)_3_X]^‐^ [X = F (**V** ^.^_F_), Cl (**V** ^.^_Cl_), Br (**V** ^.^_Br_), I (**V** ^.^_I_)]^[^ ^11^, ^18^ ^]^ and *trans‐*[Ag^III^(CF_3_)_2_X_2_]^‐^ [X = Br (**2**), Cl (**13**)] are particularly relevant,^[^ ^60^ ^]^ and a direct correlation between the number of trifluoromethyl ligands and their relative stabilities can be established. As a representative example, the stability of [Ag^III^(CF_3_)_4_]^‐^ (**IV** ^.^_CF__3_)^[^ ^17c^ ^]^ is higher than that found in the series of Ag^III^ halides, [Ag^III^(CF_3_)_3_X]^‐^ (**V** ^.^_X_; stable below 145 °C).^[^ ^11^, ^18^ ^]^ In turn, the latter complexes, [Ag^III^(CF_3_)_3_X]^‐^ (**V** ^.^_X_), revealed more stable than the corresponding dihalide anions, *trans‐*[Ag^III^(CF_3_)_2_X_2_]^‐^ (**2**, **13**).^[^ ^11^, ^18^, ^64^ ^]^ Up to now, trihalide compounds of the type [Ag^III^(CF_3_)X_3_]^‐^ remain unknown.^[^ ^58^ ^]^ Moreover, the decomposition analyses within the series of isolated anions [Ag^III^(CF_3_)_3_X]^‐^ (X = CF_3_, F, Cl, Br, I) have been studied experimentally and theoretically by Menjón and coworkers,^[^ ^11^, ^17^, ^18^ ^]^ clearly showing that Ag^III^ stability decreases with the X‐type ligand electronegativity (Figure 7). In all cases, the main decomposition pathway consists of the formal C_2_F_6_ loss through two consecutive ^•^CF_3_ radical releases.^[^ ^11^, ^18^ ^]^ A secondary decomposition pathway, negligible for the lighter halides (F, Cl) yet feasible for the heavier ones (Br, I), involves XCF_3_ release, thus contributing to their lower stability.^[^ ^18a^ ^]^ In addition, the related compounds bearing pseudo‐halides, [Ag^III^(CF_3_)_3_X]^‐^ [X = CN (**V** ^.^_CN_), N_3_ (**V** ^.^_N__3_), NO_3_ (**V** *^.^*_NO__3_)], have been recently reported.^[^ ^18^, ^61^ ^]^ Amongst them, [Ag^III^(CF_3_)_3_(CN)]^‐^ (**V** ^.^_CN_) possesses the highest stability, being similar to the one shown by [Ag^III^(CF_3_)_4_]^‐^, and in line with previous reports on the synthesis and reactivity of the cyano‐Ag^III^ anions [Ag^III^(CF_3_)_n_(CN)_4‐n_]^‐^ (n = 1–3).^[^ ^63^ ^]^ Of note, in sharp contrast to explosive Ag^I^ azides, the azido‐Ag^III^ derivative **V** ^.^_N__3_ proved remarkably stable, with thermal decomposition only occurring at temperatures above 92 °C (Figure 8).

Additional complexes of general formula [Ag^III^(CF_3_)_3_X]^‐^ [with X = SR (**V** ^.^_SR_),^[^ ^11b^ ^]^ (hetero)aryl (**V** ^.^_aryl_)^[^ ^10^ ^]^] have been characterized as transient intermediates in the reaction of [Ag^III^(CF_3_)_3_F]^‐^ (**V** ^.^_F_) with thiols,^[^ ^11b^ ^]^ in the transmetallation reaction between arylboron compounds and Ag^III^(CF_3_)_3_L (**1** ^.^_L_; L = phen, bpy),^[^ ^10a,b^ ^]^ or in the Ag^I^/Ag^II^/Ag^III^ stepwise oxidation of [Ag^I^(CF_3_)_2_]^‐^ by (hetero)aryl diazonium salts.^[^ ^10c^ ^]^ In these singular cases, the low stability of **V** ^.^_SR_ and **V** ^.^_aryl_ drove the trifluoromethylation of the thiolate^[^ ^11b^ ^]^ or aryl^[^ ^10^ ^]^ groups under mild conditions (temperature < 80 °C). For the latter case example,^[^ ^10^ ^]^ mechanistic studies confirmed that the aryl─CF_3_ bond formation took place through a concerted 2*e* ^‐^ reductive elimination pathway from **V** ^.^_aryl_, affording [Ag^I^(CF_3_)_2_]^‐^ and the corresponding trifluoromethylated arene. These studies expand the possibilities of the organic synthetic chemistry toolbox by using Ag^I^/Ag^III^ redox catalysis to promote cross‐coupling reactions similarly to palladium.

Neutral Ag^III^CF_3_ compounds are attainable by using distinct approaches. For instance, halide abstraction from [Ag^III^(CF_3_)_3_Cl]^‐^ (**V** ^.^_Cl_) using thallium salts,^[^ ^61a^ ^]^ or alternatively, the acidic treatment of [Ag^III^(CF_3_)_4_]^‐^ (**IV** ^.^_CF__3_),^[^ ^61b^ ^]^ using CH_3_CN as a solvent, give rise to the solvate complex, Ag^III^(CF_3_)_3_(NCCH_3_) (**1** ^.^_NCCH__3_). A variation of the latter procedure also applies to the formation of Ag^III^(CF_3_)_3_py (**1** ^.^_py_) in presence of pyridine (py).^[^ ^71^, ^72^ ^]^ Remarkably, starting from **1** ^.^_NCCH__3_, an array of neutral compounds Ag^III^(CF_3_)_3_L (**1** ^.^_L_; L = py, PPh_3_, AsPh_3,_ CN*t*Bu, trimethyl‐*s*‐triazine) can be obtained by facile ligand exchange.^[^ ^65^, ^71^, ^72^, ^73^ ^]^ An elegant alternative was recently reported by Baya and coworkers,^[^ ^61c^ ^]^ who unveiled the synthesis of unprecedented Ag^III^‐NHC complexes, Ag^III^(CF_3_)_3_(NHC) (**1** ^.^_NHC_; with NHC = IMes, IPr), by reacting [Ag^III^(CF_3_)_3_F]^‐^ (**V** ^.^_F_) with the corresponding imidazolium salts. Such approach enables the synthesis of Ag^III^‐NHC compounds without the need of the tedious and frequently challenging NHC isolation from imidazolium salts.

As a common feature dictated by the formally d^8^ Ag^III^ nature of these four‐coordinate silver compounds, they all exhibit *SP*‐4 geometry around the Ag^III^ center. Amongst these complexes, when isomeric conformers are possible, for instance, the plausible *cis*‐/*trans*‐isomers of [Ag^III^(CF_3_)_2_X_2_]^‐^ (**2**, **13**),^[^ ^60^ ^]^ the *trans‐*isomers are experimentally observed. DFT calculations revealed nevertheless that the *cis‐*isomers are thermodynamically favored in all cases (owing to the renowned strong *trans‐*influence of the trifluoromethyl ligand), while the *trans‐*isomers were found to be the kinetic ones. Unfortunately, the isomerization process (*trans‐* to *cis‐)* is hindered by their poor stability. Indeed, *cis‐*Ag^III^(CF_3_)_2_(edtc)^[^ ^67^ ^]^ stands for the unique *cis*‐isomer known to date, this conformation being forced by the chelate edtc ligand. Nevertheless, no XRD information is yet available for this compound, in contrast to its Cu^III^ analogue, *cis‐*Cu^III^(CF_3_)_2_(edtc).^[^ ^68^ ^]^

#### 2.5.2 Expansion of Coordination Number: Five‐ and Six‐Coordinate Ag^III^ Compounds

**Figure 9:**
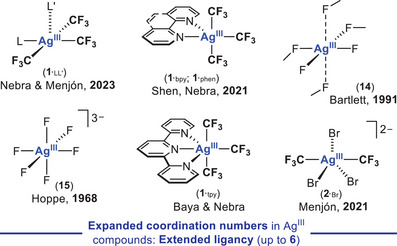
Fluorinated Ag^III^ compounds displaying *out‐of‐plane* coordination environments and an extended ligancy up to 6.

**Scheme 1:**
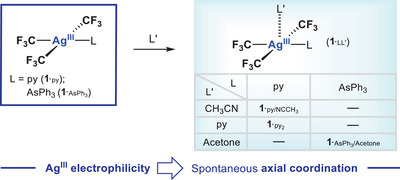
Spontaneous coordination of L‐type *N*‐donor and *O*‐donor ligands (L’ = CH_3_CN, py, acetone) to neutral Ag^III^ compounds, [Ag^III^(CF_3_)_3_L] (**1** ^.^_L_; L = py, AsPh_3_), leading to unprecedented square pyramidal Ag^III^ compounds. Proof of concept for the axial electrophilicity in *SP*‐4 Ag^III^ ions.

As commented above, the vast majority (92% of the XRD structures deposited on the CCDC database) of Ag^III^ compounds present *SP‐*4 geometry around silver, as expected for a tetracoordinate d^8^ Ag^III^ anion. Other coordination numbers and geometries can be found nevertheless. The coordination of rigid bidentate ligands seems to provoke the five‐coordination at Ag^III^ and builds Ag^III^(CF_3_)_3_(N^N) [N^N = bipyridine (**1** ^.^_bpy_), 1,10‐phenantroline (**1** ^.^_phen_); Figure 9, top center], thus displaying a square pyramidal arrangement (*SPY*‐5; *τ*<0.2) with a fairly long Ag^III…^N_ax_ interaction (>240 pm).^[^ ^10b^ ^]^ Stronger interactions have been observed in their Cu^III^ analogues, having geometries ranging from *SPY*‐5 to mostly trigonal bipyramid (*TBPY*‐5; *τ*<0.6).^[^ ^69^ ^]^ In neutral complexes of the type Ag^III^(CF_3_)_3_L [L = py (**1** ^.^_py_), AsPh_3_ (**1** ^.^_AsPh__3_); Scheme 1 and Figure 9, top left], the apical fifth coordination of an incoming neutral ligand (L’) is feasible leading to five‐coordinate *SPY*‐5 (*τ*<0.2) compounds, Ag^III^(CF_3_)_3_LL’ (**1** ^.^_LL’_; L’ = CH_3_CN, acetone, py).^[^ ^61a^ ^]^ On this occasion, the spontaneous coordination of an L‐type ligand at the apical site occurs with no geometrical imposition by the ligands. This clearly proves the tendency of Ag^III^ to extend its coordination number beyond 4, from which the existence of an axial electrophilicity in *SP*‐4 Ag^III^CF_3_ compounds is immediately inferred (see Scheme 1 below).^[^ ^10^, ^61^ ^]^ We note that the penetration index, *p*(AB), has been recently introduced by S. Alvarez^[^ ^20^ ^]^ to evaluate the magnitude of a given A^…^B contact and the interpenetration degree between the van der Waals crusts of the two involved atoms (A, B; in the current case, Ag^III^ and the *N*‐ or *O*‐donor atom of the incoming L’ ligand). This convenient descriptor *p*(AB) has two critical values, 0% (d_AB_ = v_A_ + v_B_) and 100% (d_AB_ = r_A_ + r_B_), corresponding to the limiting cases of an idealized van der Waals interaction and an idealized covalent bond, respectively. Intermediate values reflect the significance of the analyzed interactions, with indexes closer to 100% indicating stronger bonding character. With the crystallographically measured d(Ag^III^–N_ax_) (278.4 pm for **1** ^.^_py/NCCH__3_; 263.6 pm for **1** ^.^_py__2_) and d(Ag^III^–O_ax_) (265.3 pm for **1** ^.^_AsPh__3__/Acetone_) at hand, penetration indexes ranging from 70% to 76% are obtained, thereby supporting the presence of an axial acidity at neutral Ag^III^(CF_3_)_3_L platforms such as **1** ^.^_py_ or **1** ^.^_AsPh__3_, and of an efficient interpenetration between the electron clouds of the apical ligand and the Ag^III^ metal center. Of note, these findings may have relevant implications in the operating mechanism of diverse transformations mediated by Ag^III^ intermediates, and notably, within the context of Ag^III^ catalysed cross‐coupling reactions.^[^ ^12^ ^]^

On very rare occasions, a coordination number of 6 has been observed in simple binary Ag^III^ fluorides.^[^ ^7^ ^]^ Treating [Ag^III^F_4_]^‐^ (**I**) with Lewis acids affords Ag^III^F_3_ (**14**),^[^ ^7^ ^]^ one of the most powerful known oxidants.^[^ ^9^ ^]^ Structurally, the Ag^III^ in **14** is primarily surrounded by four equatorial fluorides (av. Ag^III^─F_eq_ bond length: 186 pm), while two additional bridging fluorides are located in apical positions, thus connecting contiguous Ag^III^ atoms and building a polymeric chain (av. Ag^III^─F_ax_ bond length: 199 pm; see Figure 9, top right). Moreover, two weak interactions (245 pm) are established with distinct chain fluorine atoms. Accordingly, the Ag^III^ environment in **14** can be seen as an elongated octahedral geometry (*OC‐*6), or more accurately, as square bipyramidal (*SBPY‐*6).^[^ ^7^ ^]^ A certainly ideal *OC‐*6 environment is found in the paramagnetic double perovskite Cs_2_K[Ag^III^F_6_] (**15** in Figure 9, bottom left),^[^ ^70^ ^]^ isomorphic to its Cu analogue, Cs_2_K[Cu^III^F_6_].^[^ ^71a^ ^]^

Neutral compounds of type Cu^III^(CF_3_)_3_L_3_ (L_3_ = tpy, pybox,…) have been reported by Zhang,^[^ ^72a^ ^]^ Motornov, and Ackermann,^[^ ^72b^ ^]^ independently. These unusual six‐coordinate d^8^ Cu^III^ compounds revealed diamagnetic, and thus, were characterized by NMR spectroscopy^[^ ^72^ ^]^ and Sc‐XRD^[^ ^72b^ ^]^ that evidenced a distorted octahedral (*OC*‐6) environment around Cu^III^, with *mer*‐disposition of the three trifluoromethyls. Nevertheless, to our best knowledge, the electronic structure of these Cu^III^(CF_3_)_3_L_3_ compounds has never been investigated, and a clear input behind their diamagnetism remains uncertain. Reminiscent Ag^III^ compounds displaying six‐coordination are yet unreported. As a proof of the plausible expansion of coordination number up to 6 in Ag^III^CF_3_ species, we herein anticipate that analogous Ag^III^ entities, such as Ag^III^(CF_3_)_3_tpy (**1** ^.^_tpy_; Figure 9, bottom center), can also be envisaged exhibiting a highly distorted *OC*‐6 geometry around the Ag^III^ center and diamagnetic nature, as unequivocally supported by crystallography and NMR spectroscopy on an authentic sample of **1** ^.^_tpy_, and rationalized by computational data.^[^ ^71^, ^72^ ^]^ Synthetic procedures and experimental support for the existence and magnetic behavior of **1** ^.^_tpy_ are compiled within the Ph.D. Thesis of this manuscript cofirst author (L. D.),^[^ ^65^ ^]^ and will be further disseminated elsewhere.^[^ ^64^ ^]^

A common key feature in Ag^III^ fluorides and organosilver(III) compounds containing the Ag^III^(CF_3_)_3_ fragment is their tendency to crystallize in a tetragonal symmetry (*SP*‐4, *SPY*‐5, *OC*‐6, *SBPY*‐6). The marked preference for this symmetry is reflected by the mononuclear structure of Ag^III^F_3_ (**14**), which exhibits a T‐shape structure (*C*_2v_) rather than a trigonal (*D*_3h_) arrangement.^[^ ^7^ ^]^ The structure of the hypothetical Ag^III^(CF_3_)_3_ complex also shows a T‐shape structure according to DFT calculations.^[^ ^73^ ^]^ Strikingly, a single exception to this rule is represented by the Ag^III^ tribromide complex, [Ag^III^(CF_3_)_2_Br_3_]^2‐^ (**2** ^.^_Br_ in Figure 9, bottom right), recently synthetized by Menjón by reacting *trans‐*[Ag^III^(CF_3_)_2_Br_2_]^‐^ (**2**) with PPh_4_Br.^[^ ^60^ ^]^ By contrast to the neutral compounds, Ag^III^(CF_3_)_3_LL’ (**1** ^.^_LL’_), that crystallize in *SPY‐*5 geometry, **2** ^.^_Br_ unexpectedly shows a trigonal bipyramidal environment (*TBPY*‐5, *τ*<0.71). The structures of the analogous [Ag^III^(CF_3_)_2_X_3_]^2‐^ (X = F, Cl, I) were computationally optimized and found to be *TBPY*‐5 for the Ag^III^‐Cl and Ag^III^‐I case examples, whereas [Ag^III^(CF_3_)_2_F_3_]^2‐^ shows a preference for a *SPY‐*5 geometry.^[^ ^60^ ^]^

## 3 Bonding Analysis of Ag^III^CF_3_ Compounds. ILF Electronic Structure at the Origin of the Axial Ag^III^ Electrophilicity?

From well‐established coordination chemistry principles, the basic framework for a given d^8^ TM complex is *SP*‐4 geometry. This is consistent with the large predominance of square planar arrangements observed for the vast majority of Co^I^, Rh^I^, Ir^I^, Ni^II^, Pd^II^, and Pt^II^ complexes, and also holds true for Cu^III^, Ag^III^, and Au^III^. However, a qualitative difference arises when moving from group 10 to group 11, where 2*e* ^‐^ oxidation to achieve the oxidation state +V becomes hardly accessible. That difference was first noticed by Snyder in 1995,^[^ ^74^ ^]^ who analysed the electronic structure of the homoleptic Cu^III^ anion, [Cu^III^(CF_3_)_4_]^‐^, reported only a few years before by Naumann and coworkers.^[^ ^75^ ^]^ Snyder highlighted that the frontier orbitals of [Cu^III^(CF_3_)_4_]^‐^ are mainly centered at the trifluoromethyls, whereas the molecular orbitals mainly contributed by the five 3d‐at‐metal orbitals are found well below in energy in the MO diagram. This led the author to provokingly suggest a d^10^ metal configuration, and therefore reassign its oxidation state to Cu^I^. Such statement was very controversial at the time,^[^ ^76^ ^]^ then partially forgotten, and later on brought back to the scene by Hoffmann and others,^[^ ^19^ ^]^ who also remarked the peculiar ordering of the referred five d‐at‐metal‐based molecular orbitals. These present a mirror ordering with regard to what is predicted by crystal and ligand field theory for a regular *SP*‐4 geometry, with this particular feature giving baptism to this intriguing phenomenon, ligand field inversion (ILF).

Regarding the oxidation state controversy, some authors have examined various M^III^ complexes (M = Cu, Au) using X‐ray absorption (XAS) and related core spectroscopies, sparking a heated debate about the *‘physical’* versus *‘formal’* oxidation state in such complexes.^[^ ^77^ ^]^ This scientific discussion extends far beyond the scope of this *Concept Article* and is therefore omitted herein. Furthermore, to the best of our knowledge, no such spectroscopic studies have been reported on Ag complexes thus far.

**Figure 10:**
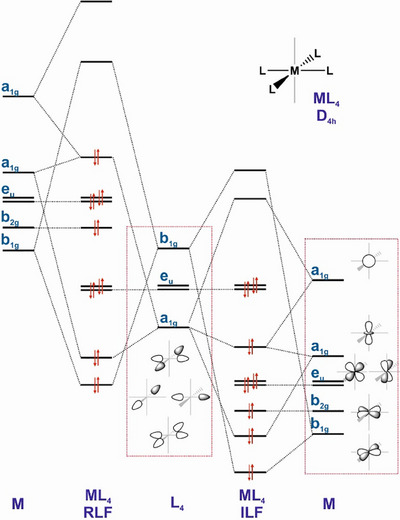
Schematic representation of the qualitative electronic structures expected for D_4h_ ML_4_ complexes showing Regular Ligand Field (RLF; left) and Inverted Ligand Field (ILF; right). For clarity, p orbitals at metal are omitted.

Regarding the electronic structure, the most general picture for a ML_4_ molecular orbital diagram ―as can be typically found in textbooks^[^ ^78^ ^]^― presents as most outstanding features a HOMO majorly showing a $d_{z^{2}}$ metal‐based character, and a LUMO showing a $d_{x^{2}-y^{2}}$ metal character. It has to be noted herein that the presence of ligands with π systems may vary this picture, although keeping most of the basic features at the resulting complexes. These account for the classical nucleophilic behavior typically found in *SP*‐4 16*e* ^‐^ TM complexes, who are therefore not prone to incorporate a fifth L‐type ligand, but to either establish dative Lewis base‐Lewis acid complexes (as M^…^M' heterobimetallic complexes),^[^ ^79^ ^]^ or alternatively, disclose 2*e* ^‐^ oxidations at the metal, giving rise to *OC*‐6, d^6^‐at‐metal compounds.^[^ ^80^ ^]^ Differently, ILF electronic structures have been found in most of *SP*‐4 Ag^III^ complexes computationally scrutinized to date.^[^ ^81^ ^]^ The implications of this phenomenon point to an expectable non nucleophilic behavior at Ag^III^, and instead, to a tendency to increase the EAN counting to 18VE. Indeed, at the origin of this ILF is the increasing stabilization of the metal valence orbitals, particularly the five d‐orbital manifold, when moving to the far right in late TMs (Figure 10). This includes the stabilization of the $d_{z^{2}}$, and also of the s and p_z_ metal orbitals, the ones that ―because of symmetry reasons― are able to take part in bonding interactions occurring along the axis perpendicular to the square coordination plane. Thus, from the MO theory point of view, the lowering of the energy of these Ag^III^ orbitals shall make the filled $d_{z^{2}}$ Ag^III^‐based molecular orbital far less available, and on the other hand, might increase the availability of the empty s/p_z_ orbitals, therefore favoring the coordination of a fifth ligand in the coordination sphere of silver. Moreover, the energies of the frontier orbitals in the studied D_4h_ Ag^III^ complexes are significantly lower than those in the isoelectronic Pd^II^ counterparts. This highlights the prominent role of the unoccupied metal orbitals in governing the electrophilic character of the referred Ag^III^ complexes, while, conversely, the occupied orbitals influence the overall reactivity of the comparable Pd^II^ compounds, which can be broadly described as nucleophilic. Consequently, two collateral effects can occur on *SP*‐4 complexes showing ILF electronic structures. On the one hand, as the frontier orbitals ―notably, HOMO and HOMO‐1― are mainly based at the ligands, reactivity on the M─L bond can be triggered by light irradiation.^[^ ^73^ ^]^ On the other hand, as empty molecular orbitals based at the metal become energetically accessible, an additional ligand coordination may occur.^[^ ^10^, ^61^ ^]^ In this general context, and even though the electronic structure of a unique D_3h_‐symmetric five‐coordinate Ag^III^ complex, [Ag^III^(CF_3_)_2_Br_3_]^2‐^ (**2** ^.^_Br_),^[^ ^60^ ^]^ has already been described and featured as an ILF case example, further studies describing this fifth interaction and the transit going from *SP*‐4 to either *SPY*‐5 or *TBPY*‐5 geometries are still missing. Thus, the MO hypothesis presented above remains to be contrasted.

**Figure 11:**
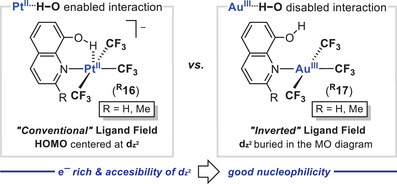
Enabled (left) versus disabled (right) M^…^H─O bonding (M = Pt^II^, Ag^III^) in isoelectronic and isoleptic [M(CF_3_)_3_(8‐hydroxyquinoline)]^n‐^ complexes [M = Pt^II^, n = 1 (**^R^16**); M = Au^III^, n = 0 (**^R^17**)] depending on their distinct electronic structures.

Besides the axial coordination of a fifth ligand in the *SP*‐4 Ag^III^ compounds **1** ^.^_L_ (L = py, AsPh_3_) facilitated by ILF (i.e., enhanced electrophilicity; Section 2.5.2),^[^ ^61a^ ^]^ an elegant study reflecting the absent axial nucleophilicity of reminiscent Au^III^CF_3_ complexes **^R^17** owing to the presence of an ILF electronic structure has been illustrated recently.^[^ ^82^ ^]^ To probe such a phenomenon, crystallographic and spectroscopic features of the Au^III^ complexes **^R^17** bearing 8‐hydroxiquinoline ligands were compared to the ones corresponding to the isoelectronic and isoleptic Pt^II^ analogues **^R^16** (Figure 11). The structural and spectroscopic parameters collected unequivocally support the presence of the Pt⋯H─O bonding. On the other hand, crystallization attempts on the organogold(III) **^H^17** systematically built adducts with HBd‐acceptors (Et_2_O or H_2_O), with the OH moiety of the quinolinyl fragment pointing out of the Au^III^ coordination environment. This markedly distinct behavior was computationally rationalized by the higher *e* ^‐^ richness of the Pt^II^ center in **^R^16** ―compared to the Au^III^ in **^R^17**― and the good availability of the mainly‐$d_{z^{2}}$ contributed HOMO in **^R^16**. Regarding the gold complexes **^R^17**, their electronic structures display an ILF. As a result, the corresponding orbital with mainly‐$d_{z^{2}}$ character is now buried in energy, rendering it not available anymore for the interaction with electrophiles.

Otherwise, within the context of trifluoromethylations, another remarkable contribution pointing to the key role of an ILF electronic configuration in the reactivity of the series of complexes [M^III^(CF_3_)_4_]^‐^ (M = Cu, Ag, Au) has been reported by the same group.^[^ ^73^ ^]^ Even though these complexes present remarkable thermal stabilities, irradiation of their solutions with UVA light causes the homolytic scission of M─CF_3_ bonds, with concomitant generation of ^•^CF_3_ radicals that can be transferred to organic molecules. This reactivity pattern is consistent with the previous discussion and supports the soundness of the calculated electronic structure for the referred set of complexes.

## 4 Summary and Outlook

Most convenient tactics enabling Ag^III^ stabilization are herein compiled, with a focus on organosilver(III) species displaying *out‐of‐plane* coordination environments and ligancy beyond 4.

The use of polydentate, most often macrocyclic, *N*‐donor ligands allows the building of an appropriate cavity and pushes the *in‐plane* coordination to host the Ag^III^ center, a key feature to enhance the stability of the resulting Ag^III^ complexes. This pioneering strategy was introduced by Rây and Chakravarty in 1943, leading to the tricationic Ag^III^ complex **3** featuring an ethylendibiguanidinium ligand (to our understanding, the unique example bearing a nonmacrocyclic polydentate *N*‐donor ligand; Figure 2, left).^[^ ^26^, ^27^ ^]^ This synthetic approach, built on coordination chemistry and ligand design principles, was followed later on by different groups allowing the preparation of porphyrin‐based Ag^III^ complexes featuring triaryl‐corroles (**^aryl^4**; Figure 2, right),^[^ ^29^, ^33^, ^34^ ^]^ NCP (**5**, **6**),^[^ ^30^, ^31^, ^32^, ^37^ ^]^ carbaporphyrins (**^R^7**)^[^ ^38^, ^39^ ^]^ and *O*‐confused porphyrins (**8**, **9**)^[^ ^41^, ^44^ ^]^ (Figure 3). More challenging resulted the coordination of flexible aryl‐tri‐aza‐macrocyclic ligands derived from the pyclen family. It was not until 2014, *ca*. 70 years after the isolation of **3**,^[^ ^26^ ^]^ when Ribas reported the oxidative addition of an aryl‐halide ligand to AgClO_4_ giving rise to the square planar aryl‐Ag^III^ complex **^H^10** (Figure 4, left).^[^ ^11a^ ^]^ This unprecedented 2*e* ^‐^ oxidation, jointly with the reactivity of **^H^10** toward nucleophiles enabling numerous aryl─heteroatom bond formations, accounts for the first clear example of 2*e* ^‐^ Ag^I^/Ag^III^ redox catalysis within the context of cross‐coupling reactions.^[^ ^11a^ ^]^ Owing to inherent ligand design, the coordination environments in these Ag^III^ platforms are restricted to square planarity.

Another major strategy finds fundamentals on the building of inorganic binary salts or homoleptic Ag^III^ anions by using X‐type, strongly electronegative ligands: that is, fluorides, oxo‐ligands, hydroxides, or imides.^[^ ^9^ ^]^ Once again, the vast majority of these compounds tend to square planarity, although the binary salts Ag^III^F_3_ (**14**) and [Ag^III^F_6_]^3‐^ (**15**) exceed coordination number 4, and crystallize in square bipyramidal (*SBPY‐*6) or octahedral (*OC*‐6) Ag^III^ environments, respectively. Besides their use as chemical oxidants,^[^ ^53^ ^]^ the applicability of this approach seems to be inhibited by its poor accessibility and handling. This is exemplified by the late isolation of **III** compared to the parent Ag^III^ compounds **3** and **^aryl^4** bearing tetra‐aza ligands and authenticated long ago.

A third strategy enabling the preparation and isolation of organosilver(III) species stems from the well‐known stabilizing effect of (per)fluoroalkyl ligands, mainly the trifluoromethyl group, when coordinated to TMs in extreme oxidation states.^[^ ^57^ ^]^ This approach was introduced by Naumann and Eujen back in the 80′s and 90′s, using toxic and hazardous CF_3_ sources, thus precluding its further development at the time.^[^ ^17^, ^63^ ^]^ Similarly to Cu^III^CF_3_ chemistry,^[^ ^83^ ^]^ a new rise of organosilver(III) chemistry saw light with the discovery of safer entries to Ag^III^CF_3_ molecular moieties by using CF_3_SiMe_3_, and either hypervalent iodine^[^ ^10^, ^17^ ^]^ or ambient air^[^ ^10b^ ^]^ as suitable oxidants to drive the Ag^I^‐to‐Ag^III^ oxidation. As a result, a large variety of *SP*‐4 Ag^III^ compounds, both neutral Ag^III^(CF_3_)_3_L (**1** ^.^_L_), and anionic [Ag^III^(CF_3_)_3_X]^‐^ (**V** ^.^_X_) or [Ag^III^(CF_3_)_2_X_2_]^‐^ (**2**, **13**), are easily available nowadays.^[^ ^10^, ^11^, ^18^, ^60^, ^61^ ^]^ As a general characteristic of these Ag^III^CF_3_ compounds, the analysis of their electronic structure evidences the presence of an ILF. This implies that the HOMO and LUMO are predominantly contributed by CF_3_ ligands, while the five 3d‐at‐metal orbitals are contributing to MOs well below in energy. This includes the filled $d_{z^{2}}$ Ag^III^‐based molecular orbital that is responsible for the observed axial nucleophilicity in some d^8^ metal systems displaying regular ligand field.^[^ ^82^ ^]^ As a consequence, the ILF electronic structure makes these *SP*‐4 Ag^III^CF_3_ platforms no longer nucleophilic, but instead, they become prone to increase the EAN count to 18VE. Definitive proof for the axial electrophilicity in neutral *SP*‐4 organosilver(III) compounds has been jointly reported by our groups, through the building of rare *SPY*‐5 compounds, Ag^III^(CF_3_)_3_LL’ (**1** ^.^_LL’_), upon spontaneous axial coordination of an L‐type ligand under no geometrical constrains (L = py, AsPh_3_; L’ = CH_3_CN, py, acetone).^[^ ^61a^ ^]^ The same type of interaction is seen in the compounds Ag^III^(CF_3_)_3_(N^N) bearing bpy or phen.^[^ ^10^, ^61^ ^]^ Another clear example of residual acidity in Ag^III^CF_3_ systems was evidenced by Menjón upon the coordination of a bromide ligand to [Ag^III^(CF_3_)_2_Br_2_]^‐^ (**2**), thereby leading to an unprecedented trigonal bipyramidal compound, [Ag^III^(CF_3_)_2_Br_3_]^2‐^ (**2** ^.^_Br_).^[^ ^60^ ^]^ Although yet unreported, the six‐coordinate complex Ag^III^(CF_3_)_3_tpy (**1** ^.^_tpy_) has been synthesized and characterized in our laboratories.^[^ ^64^, ^65^ ^]^ Akin to the series of six‐coordinate Cu^III^ complexes, Cu^III^(CF_3_)_3_L_3_,^[^ ^72b^ ^]^ **1** ^.^_tpy_ crystallizes in distorted octahedral (*OC*‐6) geometry and proved diamagnetic, markedly in contrast to the paramagnetic nature found in the double perovskite Cs_2_K[Ag^III^F_6_] (**15**),^[^ ^70^ ^]^ the only *OC*‐6 Ag^III^ compound reported to date.

**Figure 12:**
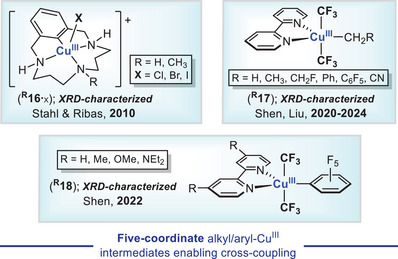
Some examples of square pyramidal Cu^III^ intermediates **^R^16** ^.^_X_, **^R^17,** and **^R^18** relevant to Ullmann‐type coupling (Stahl and Ribas, top left) and trifluoromethylation (Shen and Liu, top right and bottom).

The expansion of ligancy in *SP*‐4 organosilver(III) complexes may have relevant connotations in Ag catalysis, and notoriously, within the frame of cross‐coupling reactions operating through Ag^I^/Ag^III^ redox cycles. For instance, reminiscent five‐coordinate aryl‐Cu^III^‐X key intermediates relevant to Ullmann‐type coupling (**^R^16** ^.^_X_, R = H, CH_3_ and X = Cl, Br, I; Figure 12, top left),^[^ ^84a^ ^]^ or the trifluoromethylating competent alkyl/aryl‐Cu^III^‐CF_3_ species **^R^17** (Figure 12, top right)^[^ ^84b–d^ ^]^ and **^R^18** (Figure 12, bottom),^[^ ^84e^ ^]^ have been reported and fully characterized.

However, to our best knowledge, and despite the proposed transient life of the σ‐aryl‐Ag^III^‐X compounds **^R^10** ^.^_X_ (Figure 4, right), there is no crystallographic evidence about the participation of well‐defined five‐coordinate Ag^III^ species in cross‐coupling reactions. We thus firmly believe that future actions/directions will be taken next to further substantiate the presumed involvement of five‐ and six‐coordinate Ag^III^ compounds in C─C and C─heteroatom bond formations via 2*e* ^‐^ reductive elimination step. To this end, the exploratory use of (P,N)^C‐cyclometallated^[^ ^85a–d^ ^]^ and (P,C)^N‐hemilabile^[^ ^85e–i^ ^]^ ligands, similar to the ones successfully used in reminiscent Au^I^/Au^III^ chemistry,^[^ ^86^ ^]^ might constitute an excellent entry point to efficient Ag^I^/Ag^III^ redox catalysis, also enabling to arrest unprecedented Ag^III^ structures and geometries.

## Conflict of Interest

The authors declare no conflict of interest.

## Data Availability

The data that support the findings of this study are available in the supplementary material of this article.
